# Severe malaria in children and adolescents in Southeast Gabon

**DOI:** 10.1186/s12879-023-08133-y

**Published:** 2023-04-06

**Authors:** Judicaël Boris Lendongo Wombo, Euloge Ibinga, Sandrine Lydie Oyegue-Liabagui, Roméo Karl Imboumy Limoukou, Alain Prince Okouga, Franck Mounioko, Sydney Maghendji-Nzondo, Jean Bernard Lekana-Douki, Edgard Brice Ngoungou

**Affiliations:** 1 Department of Epidemiology, Biostatistics and Medical Informatic (DEBIM)/Research Unit in Epidemiology of Chronic Diseases and Environmental Health (UREMCSE), Faculty of Medicine, University of Health Sciences (USS), Owendo, Libreville, Gabon; 2 Center of Epidemiology, Biostatistics, and Research Methodology-Gabon (CEBIMER-Gabon), Higher Institute of Medical Biology (ISBM), University of Health Sciences (USS), Owendo, Libreville, Gabon; 3 Unit of Evolution, Epidemiology and Parasite Resistance (UNEEREP), Franceville Interdisciplinary Center for Medical Research (CIRMF), Franceville, Gabon; 4 Central African Regional Doctoral School in Tropical Infectiology (ECODRAC), Franceville, Gabon; 5 Centre Interdisciplinaire de Recherches Medicales de Franceville (CIRMF), Université des Sciences de la Santé (USS), Libreville, Gabon; 6 Department of Biology, Faculty of Science, Masuku University of Science and Technology (USTM), Franceville, Gabon; 7 Department of Parasitology-Mycology, University of Health Sciences (USS), Libreville, Gabon

**Keywords:** Severe malaria, Children, Malaria prevalence, Severe forms

## Abstract

**Introduction:**

Malaria remains a significant public health problem in sub-Saharan Africa. Child mortality due to severe malaria remains high in developing countries despite improvements in malaria management and a better understanding of its pathophysiology. To address the lack of epidemiological studies on severe malaria in Gabon, this study describes the epidemiological aspects of severe malaria in rural, semi-rural, and urban areas of southeast Gabon.

**Methods:**

Demographic, clinical, and laboratory data for children and adolescents aged 0–18 years were collected in 2019 from hospital records at three health facilities in southeastern Gabon. The patients included in the study were positive for *P falciparum* malaria diagnosed by microscopy with at least one of the malaria severity criteria.

**Results:**

Severe malaria accounted for 18.8% (667/3552) of malaria cases. Children aged 0–5 years accounted for 71.8% (479/667) of all severe malaria cases. Adolescents over 15 years of age were the least affected by severe malaria with 4.2% (28/667). Across the study, severe anemia (49.0%, 327/667), convulsions (43.0%, 287/667), respiratory distress (5.1%, 34/667), and altered consciousness (4.8%, 32/667) were the most frequent clinical signs of severe malaria in children. Franceville was the locality most affected by severe malaria with 49.2% (328/667), followed by Koulamoutou with 42.0% (280/667) and Lastourville with 8.8% (59/667). Convulsions (50.6%, 166/328) and coma (6.1%, 20/328) were more frequent in children living in urban areas. In contrast, severe anemia (56.7%, 186/339) and jaundice (6.8%, 23/339) were more common in children living in semi-rural areas.

**Conclusion:**

Severe malaria is more prevalent in urban areas in regions with a high malaria transmission intensity. However, in this study, the epidemiological characteristics of severe malaria were similar in the three settings (urban, rural, and semi-rural areas) despite different levels of urbanization. Nevertheless, the various signs of severity were more frequent in Franceville, an urban area. Children under 5 years of age remain the most vulnerable age group.

## Introduction

Despite control efforts, malaria remains a significant public health problem in sub-Saharan Africa. According to the World Health Organization (WHO), in 2020, 241 million cases of malaria and 627,000 deaths were recorded worldwide, 77% of which affected children under 5 years of age. That same year, 96% of all malaria cases were recorded in sub-Saharan Africa [1]. Malaria is a disease caused by parasitic protozoa of the genus *Plasmodium*. The clinical presentations of malaria may vary and include asymptomatic malaria, uncomplicated malaria, or severe malaria (Newton and Krishna, 1998). The species *Plasmodium falciparum* is most often responsible for severe forms of malaria [2]. Severe malaria is defined by the presence of *Plasmodium falciparum* in the blood and is associated with one or more clinical signs [3]. These include neurological complications (e.g., in the case of human cerebral malaria), severe anemia, respiratory distress, hypoglycemia, and metabolic disorders (Clarke et al., 2017). Child mortality due to severe malaria remains high in developing countries despite improvements in malaria management and a better understanding of its pathophysiology [4, 5]. Moreover, in areas of high malaria transmission, the distribution of malaria cases is not homogeneous [6–8].

In Gabon, a Central African country composed of 9 provinces, there is a high intensity of malaria transmission throughout the year. The entomological inoculation rate can reach approximately 1,000 bites of infected *female anopheles* per person [9]. Disparities in the distribution of infection exist between rural, semi-rural and urban environments [6, 8, 10]. Moreover, malaria is one of the main causes of consultation in hospital emergency wards [11–13].

In Gabon, the main signs of severe malaria in children are severe anemia, neurological complications, hypoglycemia and respiratory distress. In children under 5 years of age, severe anemia is the most frequent clinical sign in severe forms [12, 14–16]. Previous studies have also reported that older children (and even adults) who would normally have acquired immunity may develop severe forms of the disease [15, 17]. However, only few studies have been conducted on the epidemiological aspects of severe malaria in rural and semi-rural areas in the country. Hence, the objective of this study is to describe the epidemiological aspects of severe malaria in rural, semi-rural, and urban areas of southeastern Gabon.

## Materials and methods

### Location of the study

This retrospective study was conducted in three localities in southeastern Gabon in 2019. Franceville is the administrative capital of the Haut-Ogooué province (1°37′15″S and 13°34′58″E). It is an urban area and the third largest city in the country. Koulamoutou, the administrative capital of the Ogooué-Lolo province (1°13′10″S, 12°28′0″E), is a semi-urban area. Finally, Lastourville is the capital of the Mulundu department and a rural region of Gabon (0°49′S, 12°42′E). The records used in this study came from the Amissa Bongo Regional Hospital of Franceville, the Paul Moukambi Hospital of Koulamoutou, and the outpatient department of the medical center of Lastourville.

### Study population and data collection

This retrospective study targeted patients aged 0 to 18 with severe malaria. Data collection was conducted in the same manner in the three health facilities. A standardized sheet was use for data collection from the hospital registers. All socio-demographic, biological and clinical information have been collected.

### Severe malaria definition

Severe malaria was defined by the detection of asexual *P. falciparum* parasitaemia by microscopy with at least one of the criteria for severe malaria according to the WHO definition : Altered consciousness (GCS score 9–14 or Blantyre score 3–4 in children under 5 years of age), coma (with a Blantyre score < 3 in children under 5 years of age and a GCS score < 9 in older children), respiratory distress (presence of abnormalities in respiratory rate, rhythm (Kussmaul or Cheyne-Stokes breathing)), Multiple convulsions (More than one seizure in 24 h), Prostration (generalized weakness so that the patient is unable walk or sit up without assistance), shock (PAS < 70 mm Hg in children or < 80 mm Hg in adults), pulmonary edema (Radiologic diagnosis), abnormal bleeding (Gingivorrhagia, epistaxis, hematemesis, melena, or prolonged bleeding from venipuncture sites), jaundice (bilirubinemia > 50 µmol (3 mg / dl) with a parasite count > 100 000 / µl), severe anemia (Hb < 5 g/dl or Ht < 15% in children < 12 years and Hb < 7 g/dl or Ht < 20% in children > 12 years and adults), hypoglycemia (Blood glucose or plasma glucose < 2. 2 mmol (< 40 mg / dl)), Hyperparasitemia (P. falciparum parasitemia > 10%), hyperlactatemia (lactate > 5 mmol/l), Renal failure (Creatininemia > 265 µmol (3 mg/dl) or blood urea > 20 mmol), metabolic acidosis (Base deficit > 8 mEq / l or plasma bicarbonate < 15 mmol or venous plasma lactate > 5 mmol), identified by WHO.

Data were collected from medical and hospital records dating from 2016 to 2018. This study analyzed records of minor patients who consulted for malaria in emergency and outpatient departments. Adult, non-malarial patients were not included. Clinical and demographic data (age and sex) of patients were collected from the emergency department records of each health facility. In addition, patients’ biological data were collected from laboratory records.

In the health facilities of the region, malaria is diagnosed by optical microscopy. The thick drop examination is positive if asexual forms of *Plasmodium* have been observed by at least two microscopists and the parasitemia is expressed qualitatively, as follow: low (+) 1–10/100 fields, light (++) 11–100/100 fields, moderate (+++) 1–10/one field, and high parasitemia (++++) > 10/one field [18]. These scores were used to estimate parasite densities: + = 10 to 90 parasites/µl; ++ = 100 to 1,000 parasites/µl, +++ = 1,000 to 10,000 parasites/µl; ++++ = >10,000 parasites/µl, assuming a white blood cell count of 8,000/µl [19].

### Statistical analysis

All collected data were initially recorded on a spreadsheet and analyzed using the Stata software (version 12.0, Stata Corporation, College Station, Texas, USA).

Variables were described using proportions for qualitative variables, means and standard deviation for quantitative variables. Proportions were compared using the Chi2 test or Fisher’s exact test. The significance level was set at α = 5% for all analyses. All patients with missing data were excluded from the analysis.

### Ethical and regulatory considerations

The consultation of laboratory records was performed in accordance with the Declaration of Helsinki 2000. This study was approved by the National Research Ethics Committee of Gabon (N°001/PR/SG/CNER/2018) which waives the informed consent of the patient in the framework of a retrospective study and by the management of the CHRAB of Franceville, CHRPM of Koulamoutou and CM of Lastourville, guarantor of the hospital data. The data were collected anonymously as part of the routine analyses carried out in the laboratories of these health structures. These facilities are under the supervision of a Director.

## Results

### Demographic characteristics of included patients

In total, 6114 febrile children and adolescents consulted the different study sites. Among these, 58.1% (3552) patients tested positive for *Plasmodium falciparum* by microscopy (thick drop) and 18.8% (667/3552) had severe malaria. Table 1 shows that, of the 667 patients who developed at least one of the signs for malaria severity, the proportion of male 53.4% (355/665) and female 46.6% (310/665) children was approximately the same. The sex ratio (males to females) was 1.1. The mean age of the patients was 6 ± 4.96 years. Children aged 0–5 years accounted for about 71.8% (479) of severe malaria cases. The children in the [6–10 years] age group accounted for 18.6% (124) of cases. Patients older than 15 years had the lowest proportion of cases with 4.2% (28). Franceville had the highest proportion of severe malaria cases with 49.2% (328), followed by Koulamoutou with 42.0% (280) of cases (Table 1).

**Table 1:** Demographic characteristics of patients with severe malaria

Characteristics	Number (n)	Proportion (%)
**Sex**		
Male	355	53.4
Female	310	46.6
**Age groups (in years)**		
[0–5]	479	71.8
[6–10]	124	18.6
[11–15]	36	5.4
> 15	28	4.2
**Study site**		
Franceville (CHRAB)^*^	328	49.2
Koulamoutou (CHRPM)^**^	280	42.0
Lastourville (CM)^***^	59	8.8

^*^CHRAB: Centre Hospitalier Régional Amissa Bongo, ^**^CHRPM: Centre Hospitalier

Régional Paul Moukambi and ^***^CM: Centre Médical

### Characteristics of severe malaria in the study population

In this study, severe anaemia (49.0%), convulsions (43.0%), respiratory distress (5.1%) and altered consciousness (4.8%) were the most frequently observed clinical signs of severe malaria. Jaundice (3.7%), coma (3.4%), hypoglycaemia (0.6%) and renal failure (0.4%) were the least severe forms observed (Table 2).

**Table 2:** Description of the clinical signs of severe malaria in southeastern Gabon

Severe forms	Number (N = 667)	Percentage
**Clinical data**		
Coma	23	3.4
Altered consciousness	32	4.8
Convulsions	287	43.0
Respiratory distress	34	5.1
Jaundice	25	3.7
**Laboratory data**		
Severe anemia	327	49.0
Hypoglycemia	4	0.6
Renal failure	3	0.4

### Distribution of severe signs by age group

Table 3 shows the distribution of severe malaria according to each age group. In children aged 0–5 years, severe anaemia (49.3%, 236), convulsions (46.1%, 221) and respiratory distress (5.4%, 26) were the most frequently encountered clinical signs. In the 6–10 years age group, severe anaemia (54.0%, 67), convulsions (39.5%, 49), coma (4.0%, 5) and altered consciousness (4.0%, 5) were the most frequent clinical signs of severe malaria. Among the age groups, the age group of children under 5 years had the highest proportions of patients affected by seizures (221), jaundice (14) and severe anemia (236) compared to the age groups (p < 0.05). The age group of adolescents over 15 years had the highest proportion of patients with renal failure (2) (p < 0.01). The majority of patients had a parasite load greater than 10,000 parasites/µl of blood. Children aged 0–5 years had the highest parasite loads and adolescents over 15 years had the lowest parasite loads p < 0.001.

In 3.7% of severe cases, we observed an association between at least one neurological sign and severe anaemia (haemoglobin level < 5 g/dL in children and haemoglobin level < 7 g/dL in minors over 12 years of age).

**Table 3:** Distribution of bioclinical characteristics of severe malaria according to age groups

Severe cases	Age groups (year)				*P*-value
	[0–5] (n = 479)	[6–10] (n = 124)	[11–15] (n = 36)	> 15 (n = 28)	
**Clinical data**					
Coma, n (%)	12 (2.5)	5 (4.0)	5 (13.8)	1 (3.6)	0.052
Altered consciousness, n (%)	14 (3.0)	5 (4.0)	6 (16.6)	7 (25)	0.36
Convulsions, n (%)	221 (46.1)	49 (39.5)	8 (22.2)	9 (32.1)	**0.02**
Respiratory distress, n (%)	26 (5.4)	4 (3.2)	1 (2.8)	3 (10.7)	0.33
Jaundice, n (%)	14 (3.0)	4 (3.2)	4 (11.1)	3 (10.7)	**0.04**
**Laboratory data**					
Severe anemia, n (%)	236 (49.3)	67 (54.0)	20 (55.5)	4 (14.3)	**< 0.01**
Hypoglycemia, n (%)	2 (0.4)	1 (1.0)	1 (2.8)	0 (0)	0.27
Renal failure, n (%)	1 (0.2)	0 (0)	0 (0)	2 (7.1)	**< 0.01**
Parasitemia laod (p*/µl)					
**++**, n (%)	13 (26.0)	35 (70.0)	1 (2.0)	1 (2.0)	**< 0.001**
+++, n (%)	175 (88.4)	12 (6.1)	6 (3.0)	5 (2.5)	
++++, n (%)	291 (69.4)	77 (18.4)	29 (6.9)	22 (5.3)	

*P: parasites

### Biological characteristics patients with severe malaria

Table 4 shows the distribution of laboratory parameters according to age group. Children aged 0 to 5 years had a mean low hemoglobin level 8.3 ± 1.8 g/dL, and a high platelet level 285.3 ± 179.1 103/µL. Children older than 15 years had a high hemoglobin level 10.6 ± 1.5 g/dL, and the platelet level low 120.0 ± 68.9 103/µL.

**Table 4:** Distribution of biological characteristics according to patient age groups

Characteristics	Age groups (year)			
	[0–5]	[6–10]	[11–15]	> 15
Haemoglobin (g/dL)	8.3 ± 1.8	8.9 ± 1.9	9.6 ± 2.5	10.6 ± 1.5
Haematocrit (%)	30.1 ± 7.4	31.8 ± 7.4	31.3 ± 10.1	38.3 ± 4.9
Platelets (10^3^/µL)	285.3 ± 179.1	221.5 ± 139.9	192.5 ± 171.9	120.0 ± 68.9
White blood cells (10^3^/µL)	10.2 ± 4.1	10.4 ± 4.0	9.2 ± 4.2	7.7 ± 1.2
Red blood cells (10^6^/µL)	3.9 ± 0.7	3.5 ± 1.5	3.8 ± 1.4	4.8 ± 0.3

### Distribution of severe malaria and deaths according to site

The distribution of severe forms of malaria in Franceville (328 severe cases), Koulamoutou (280 severe cases) and Lastourville (59 severe cases) is presented in Table 5. Coma (6.4%, 20/328) and convulsions (50.6, 166/328) were more frequent in Franceville compared to Koulamoutou (1.1%, 3/280; 36.4%, 102/280) and Lastourville (0%, 0/59; 32.2%, 19/59) with a stastically significant difference (p < 0.05). Severe anemia (55.0%, 154/280) and jaundice (8.2%, 23/280) were more frequent in Koulamoutou compared to Franceville (43.0%, 141/328; 0.6%, 2/328) and Lastourville (54.2%, 32/59; 0.0%, 0/59) with a stastically significant difference (p < 0.05). Lastourville was the site most affected by respiratory distress (10.2%, 6/59) compared to Franceville (6.4%, 21/328) and Koulamoutou (2.5%, 7/280) (p = 0.01). The analysis did not reveal any significant difference between the different site for altered consciousness, hypoglycemia and renal failure. Severe malaria was less prevalent in Lastourville (Table 5).

**Table 5:** Distribution of severe malaria criteria according to site

	Franceville	Koulamoutou	Lastourville	*p*-value
Coma, n (%)	20 (6.1)	3 (1.1)	0.0 (0)	< 0.01
Altered consciousness, n (%)	15 (4.6)	16 (4.9)	1 (1.7)	0.42
Convulsions, n (%)	166 (50.6)	102 (36.4)	19 (32.2)	< 0.001
Respiratory distress, n (%)	21 (6.4)	7 (2.5)	6 (10.2)	0.01
Severe anemia, n (%)	141 (43.0)	154 (55)	32 (54.2)	0.02
Jaundice, n (%)	2 (0.6)	23 (8.2)	0.0 (0)	< 0.001
Hypoglycemia, n (%)	3 (0.9)	1 (0.4)	0.0 (0)	0.54
Renal failure, n (%)	2 (0.6)	0.0 (0)	1 (1.7)	0.17

### Comparison of severe forms and malaria deaths between urban and semi-rural areas

Severe malaria was divided between urban (328 severe cases observed) and semi-rural (339 severe cases) settings. Table 6 shows that coma and convulsions were more frequent in children from urban areas [6.1% (20/328) and 50.6% (166/328), respectively] than in children from semi-rural areas (p = 0.0005 and p = 0.00008, respectively). Severe anemia (56.7%, 186/339) and jaundice (6.8%, 23/339) were more prevalent in children from semi-rural areas compared to children in urban areas (p = 0.007 and p = 0.00007, respectively). No significant differences were observed for altered consciousness, respiratory distress, hypoglycemia, and renal failure (p > 0.05) between the two settings. Although no significant difference in malaria-related deaths was observed between children living in the two areas (p = 0.27), children living in the semi-rural area had a higher number of deaths. The severe forms associated with death were predominantly altered consciousness (9.4%; 3/32), followed by respiratory distress (5.9%; 2/34), coma (4.3%; 1/23) and severe anemia (3.7%; 12/327).

**Table 6:** Comparison of severe malaria between children in urban and semi-rural areas

Severe cases	Urban area FCV	Semi-rural area KM-LTV	*p*-value
Coma, n (%)	20 (6.1)	3 (1.0)	< 0.01
Altered consciousness, n (%)	15 (4.6)	17 (5.0)	0.96
Convulsions, n (%)	166 (50.6)	121 (35.7)	< 0.001
Respiratory distress, n (%)	21 (6.4)	13 (3.8)	0.17
Severe anemia, n (%)	141 (43.0)	186 (56.7)	< 0.01
Jaundice, n (%)	2 (0.6)	23 (6.8)	< 0.001
Hypoglycemia, n (%)	3 (1.0)	1 (0.3)	0.36
Renal failure, n (%)	2 (0.6)	1 (0.3)	0.62
Deceased, n (%)	6 (1.8)	12 (3.5)	0.271

## Discussion

There is a lack of data on severe forms of malaria in sub-Saharan Africa, a form of the disease which is responsible for important morbi-mortalities, especially in children. The objective of this study was to describe the epidemiological aspects of severe malaria in children and adolescents in southeast Gabon. The choice of the study period was motivated by the observation of a rebound in malaria prevalence in Gabon after 2010 [20–22]. The retrospective nature of this work restricted the sample size to health records available for the study period. In addition, this study was challenged by the difficulty of locating and gathering patient records from different laboratory and emergency departments in the various health centers. During the data collection period, the three health facilities received 3552 children and adolescents with malaria in their emergency departments, including 667 cases with signs of severe malaria representing a prevalence of 18.8%. This prevalence is close to that observed between 2000 and 2002 at Libreville (18.4% of severe cases) and lower than that (26.15%) observed in Cameroon [23, 24]. The majority of severe malaria cases occurred in children aged 0–5 years (71.8%, 479/667), followed by children aged 6–10 years (18.6%, 124/667). This result is in line with the WHO, which states that children under 5 years of age are the most vulnerable to malaria [25]. Among all the severe malaria cases, the proportion of males (53.4%) appears to be higher than that of females (46.6%). Similar observations have been made in other populations of hospitalized children in Gabon and in other countries [6, 24, 26, 27].

Severe anemia, convulsions, coma and respiratory distress were the most severe forms observed. These results are similar to those observed in other studies [23, 24, 26, 28, 29]. Children under 5 years of age were the most affected by severe forms of malaria infection. These results could be explained by the fact that children under 5 years of age remain a population at risk for malaria [6, 23]. Indeed, children under 5 years of age living in areas of high malaria transmission have immature antimalarial immunity. This presentation of severe malaria reflects the epidemiology of Plasmodium infection in areas of high transmission. In this same age group (children under 5 years of age), mean parasitemia was highest, and mean hemoglobin and hematocrit levels were lowest. In contrast, adolescents over 15 years of age had renal failure, a severe form of malaria often seen in those over 15 years of age [30]. This result could be explained by malnutrition in children. Furthermore, studies have shown that in high transmission areas such as Gabon, the acquisition of antimalarial immunity begins after the age of 5 years [30–33].

In our study, the majority of children had a parasitaemia of more than 10,000 parasites/µl of blood. The severe form hyperparasitaemia was not observed. This result could be explained by the fact that the parasite load was estimated qualitatively and not quantitatively. However, although hyperparasitaemia is considered a severity criterion for malaria, the presence of P. falciparum parasitemia associated with at least one severity criterion is considered severe malaria, according to the WHO [3, 34]. Moreover, in hyperendemic areas, sequestration of P. falciparum-infected red blood cells is the fundamental pathological process of P. falciparum malaria, leading to misestimation of the true parasitaemia [35, 36].

However, Franceville had the highest prevalence of severe malaria compared to the other sites. These severe forms of malaria infection were significantly different between the three areas (*p* < 0.05), except for altered consciousness, respiratory distress, hypoglycemia, and renal failure. The low proportion of patients in Lastourville could be at the origin of this difference observed between sites. The difference in the prevalence of these severe forms between sites (urban, semi-rural and rural) could be explained by the fact that hospital facilities are more accessible in Franceville than in the other sites. Moreover, there is also a difference in the density of the resident child population in each site associated with economic hardship which restricts prompt access of patients and families to a health facility. Similar results were found in other studies conducted in Gabon [7, 10]. A similar study in Uganda showed that the clinical features of severe malaria may differ between urban and rural hospital facilities [37].

In urban areas, the proportion of severe malaria was higher than in semi-rural areas. This result could be explained by the fact that children living in urban areas have malaria immunity acquired later due to the low intensity of malaria transmission, unlike in semi-rural and rural areas. These results are similar to those of Issoufou et al. in 2007. The prevalence of convulsions and coma were significantly higher in urban areas than in semi-rural areas (p < 0.05). In addition, the prevalence of severe anemia and jaundice were significantly higher in semi-rural areas than in urban areas (p < 0.05). This result is similar to that observed in Equatorial Guinea [38]. However, it is contrary to the results of Issoufou in 2007, where severe anemia was more observed in children living in urban areas (Issifou et al., 2007).

In this study, the mortality rate was 2.7%. This rate was close to that observed by Isoufou et al. in Lambaréné (center of Gabon) and by Andreas Chiabi and colleagues (3.8%) in Cameroon [23, 39].

This could be explained by the fact that the Koulamoutou CHRPM is the reference hospital in the Ogooué-Lolo province. Cases of severe malaria from other health facilities in the region are systematically transferred to the CHRPM. For example, serious cases of malaria from the Lastourville Medical Center, a secondary health establishment within the same health region, are transferred to the Koulamoutou CHRPM, which is better equipped to treat serious cases. This result could also be explained by the practice of self-medication by parents [7, 21, 40]. Considering the type of environment, the proportion of deaths observed in urban areas was not significantly different from that observed in semi-rural areas (*p* > 0.05). This result could be explained by the existence of road infrastructures between a large number of villages and localities with a hospital structure, facilitating access to hospitals. In addition, malaria control measures across the country could explain this lack of difference in the fatality rates. The various severe forms associated with death were: altered consciousness, followed by respiratory distress and coma. These results are similar to those observed in other studies where the occurrence of coma, altered consciousness and respiratory distress were associated with death [12, 39, 41, 42].

### Limitation

This retrospective study has a few limitations. This retrospective study did not allow us to find in the registers all the criteria of the definition of severe malaria, in particular, shock, pulmonary edema, abnormal bleeding, metabolic acidosis and hyperlactatemia [3]. The Blantyre and Glasgow scores that accompany the clinical diagnosis of coma and altered consciousness are not mentioned in the register. The absence of a quantitative estimate of the parasite load was also a limit to our study.

## Conclusion

The epidemiological characteristics of severe malaria were similar in the two settings (urban and semi-rural), despite different levels of urbanization. However, the proportions of severe malaria cases were higher in Franceville, which is an urban area. Children aged 0–5 years were the most affected by severe malaria and deaths due to this disease. Coma, convulsions, severe anemia, jaundice, and respiratory distress were the most common severe clinical signs. Severe malaria was more common in children living in the urban area than in those living in the semi-rural area. Furthermore, the death rate in these two types of settings was similar. However, these observations may underestimate the real prevalence of severe malaria in southeastern Gabon. Indeed, the poor state of health records and the loss of medical records have largely contributed to the loss of patient information. It is therefore necessary to characterize severe malaria in all regions of the country to gain a better idea of the national prevalence of severe malaria and its characteristics in Gabon.

## Data Availability

Data sets used and/or analyzed during the current study are available from the corresponding author upon reasonable request.

## List of abbreviations

WHO: World Health Organization
*P. falciparum*: *Plasmodium falciparum*
CHRAB: Centre Hospitalier Regional Amissa Bongo
CHRPM: Centre Hospitalier Regional Paul Moukambi
CM: Centre Médical
FCV: Franceville
KM: Koulamoutou
LTV: Lastourville
